# No evidence for a dilution effect of the non-native snail, *Potamopyrgus antipodarum*, on native snails

**DOI:** 10.1371/journal.pone.0239762

**Published:** 2020-10-01

**Authors:** Michele D. Larson, Edward P. Levri, Snehalata V. Huzurbazar, Daniel J. Greenwood, Kara L. Wise, Amy C. Krist

**Affiliations:** 1 Department of Zoology & Physiology, Program in Ecology, University of Wyoming, Laramie, Wyoming, United States of America; 2 Division of Mathematics and Natural Science, Penn State Altoona, Altoona, Pennsylvania, United States of America; 3 Department of Biostatistics, West Virginia University, Morgantown, West Virginia, United States of America; Instituto Federal de Educacao Ciencia e Tecnologia Goiano - Campus Urutai, BRAZIL

## Abstract

The dilution effect can occur by a range of mechanisms and results in reduced parasite prevalence in host taxa. In invaded ecosystems, the dilution effect can benefit native species if non-native species, acting as resistant or less competent hosts, reduce rates of parasitic infections in native species. In field experiments, we assessed whether manipulating biomass of the non-native snail, *Potamopyrgus antipodarum*, caused a dilution effect by reducing trematode infections in three taxa of native snails. In contrast to many studies showing resistant or less competent non-native hosts can “dilute” or reduce infection rates, we found no evidence for a dilution effect reducing infection rates of any of the native snails. We suggest that a dilution effect may not have occurred because most trematode taxa are highly host specific, and thus the trematode transmission stages did not recognize the invasive snail as a possible host. In this case, community composition appears to be important in influencing the dilution effect.

## Introduction

Interactions between parasites and non-native and native hosts can have diverse outcomes. Non-native species can bring novel parasites to native hosts [1–3], serve as reservoirs for native parasites [1, 2], or reduce infection rates in a native species by acting as resistant, less competent, or decoy hosts (hereafter: less competent hosts) [1, 2, 4, 5]. The latter results in a “dilution effect.” The dilution effect, a reduction of parasite prevalence with increasing host diversity, can be caused by diverse mechanisms broadly ranging from changes in native biodiversity to the introduction of non-native species [1, 5]. The effects of a non-native on parasite prevalence in the primary host can be direct or indirect. In parasites with free-living transmission stages, the most direct effect occurs when the parasite transmission stages are removed from the environment by penetration of a less competent host, consumption by a predator, or through chemical or physical interference with the transmission stage [4, 6–12]. Direct effects of the dilution effect reducing trematode parasites are widespread. For example, non-native American slipper limpets and Pacific oysters reduce the number of trematodes per infection (infection load) in a native mussel by serving as an alternative, less competent target for a trematode transmission stage [6] and a non-native snail can reduce trematode prevalence (infection rate) in a native snail by consuming parasite eggs [7]. Bivalves, barnacles, shrimp, crabs, amphipods, and sea anemones can directly consume trematode cercaria [8, 11, 12]. Algae and seaweed can physically obstruct cercaria [11–13], whereas crabs and shrimp can consume trematode larvae or physically interfere with larvae transmission [11]. Prevalence of the human trematode parasite, *Schistosoma mansoni*, in snail hosts can be reduced by planaria or by planaria-derived excretions and mucus, alone, suggesting that the exudates alone can kill or disorient trematode larvae from finding snail hosts [9].

The diversity and species composition of a community can also indirectly affect whether the dilution effect occurs (i.e. reduce the parasite prevalence in a primary host species) [14–16]. For example, more diverse communities can have fewer diseases and parasites [14], and increased diversity of vertebrate hosts can decrease disease prevalence in mammals that are hosts of the tick-transmitted bacterium which causes Lyme Disease [17, 18]. Prevalence of Lyme Disease falls with increasing proportions of incompetent and non-transmitting hosts (squirrels and shrews; [18]). In general, increasing species richness in a community can reduce prevalence in the most competent hosts by 1) reducing relative abundance of the intended host [5, 14, 19], 2) causing changes in the behavior of the host or parasite, or 3) by altering the condition of the host [5, 19].

The dilution effect could play an important role in invasion ecology because non-native species may reduce prevalence of infection in native species by removing parasite transmission stages that could otherwise infect natives. This can occur if the parasites “perceive” the non-native as a potential target, and the non-native is a less competent host than the native [6, 7, 20–23]. Multiple native fish showed substantial decreases in *Diplostomum* trematode infection in the St. Lawrence River with the invasion of the round goby [24], and an increase in the abundance of the invasive bank vole resulted in reduced prevalence of infection by *Bartonella* bacteria in two species of wood mouse in Ireland [21]. In addition, in Hawai’i, nematode infection rates of native and introduced freshwater fishes were lower in sympatry compared to allopatry [25].

In New Zealand, the freshwater snail, *Potamopyrgus antipodarum*, is parasitized by at least 18 different trematodes [26] with some populations reaching 80% prevalence (infection rates) [27]. Yet, *Potamopyrgus* is very rarely infected with trematodes where it is non-native [28–31], suggesting that it may be a less competent host to native trematodes. *Potamopyrgus* also has the potential to cause a dilution effect for native snail species because it can occur at extremely high densities where it occurs as a non-native (> 600,000 ind/m^2^) [32]. We conducted field experiments to determine whether increasing biomass of *Potamopyrgus* could reduce rates of trematode parasitism in three taxa of native snails. Because native snails are frequently infected by castrating trematodes [29, 31], reductions in the prevalence of trematodes in native snails could have substantial demographic benefits when prevalence rates are consistently high. If *Potamopyrgus* dilute trematode infection rates of native snails, we should observe decreasing trematode prevalence in native snails with increasing biomass of the non-native snail.

## Methods and methods

### Study site

We conducted field experiments in Polecat Creek in the John D. Rockefeller Jr. Memorial Parkway in northwest Wyoming. Our study site is just south (within view) of the bridge on Grassy Lake Road, approximately 2 km west of Flagg Ranch Resort (UTM coordinates: 12T 525215m E, 4883969m N). Polecat Creek is a geothermally fed stream with relatively stable summer flows (0.25–0.35 m/s; M. Larson, unpublished data). The stream channel is roughly 16 m wide and entrenched (steep vertical drop-off from shore to creek bottom of approximately 0.5–1 m) with discharges of < 2 m^3^/s (M. Larson, personal observations) [33]. In July 2015, the mean temperature was 20.0º C (+/- 2.13) and mean specific electrical conductivity was 158.6 μS/cm (+/- 15.0). The streambed at our study site was gravel with large beds of macrophytes. We conducted all field research following guidelines from Grand Teton National Park (Permit GRTE-2014-SCI-0039 and GRTE-2015-SCI-0042).

### Snail and trematode assemblages

Five native snails co-occur with *Potamopyrgus* at our study site in Polecat Creek. A single species of the family Physidae (*Physa* sp.) and the family Lymnaeidae (*Galba* sp.) occur at moderate densities (S1 Table, M. Larson, personal observations). Two other snails (Planorbidea: *Gyraulus* sp. and *Planorbella* sp.) are less abundant and usually occur along the shoreline on submerged macrophytes (M. Larson, personal observations). These four species are all pulmonates (air breathing snails). One native snail, *Pyrgulopsis robusta*, and the non-native snail, *Potamopyrgus*, occur at much higher densities at the field site (S1 Table, M. Larson, personal observations) and are prosobranchs (gill breathing snails) with harder shells and smaller adult body sizes than the pulmonate snails. Densities of all of these species vary from year to year and can be patchily distributed at and near the study site in Polecat Creek (S1 Table). Except for *Gyraulus*, all the native snails at our study site are hosts to trematode parasites [29, 31]. For our field experiments, we assessed the effect of biomass of the non-native snail *Potamopyrgus* on trematode infection rates in the three most common native snails *Physa*, *Galba*, and *Pyrgulopsis*.

Trematodes use multiple hosts to complete their life cycles [34, 35]. In freshwater ecosystems, snails (or bivalves) are the first intermediate host and are infected by either penetration of a free-swimming larval stage (miracidium) or by ingestion of an encapsulated, sessile embryo [34, 35]. After development of multiple stages and extensive asexual reproduction within the snail host, free-swimming transmission stages (cercariae) exit the snail host and directly penetrate the final or definitive host, encyst in the environment, or encyst in a second intermediate host [34, 35]. Both free-swimming larval stages (miracidia and cercaria) are short lived in the environment with most species surviving < 24 hours at temperatures between 20-25º C [34, 36, 37]. No further development or replication of the trematode occurs in the second intermediate host, which can be a diverse array of animals ranging from zooplankton, insect larvae, amphipods, and mollusks, to fish and amphibians [34, 35]. The definitive, or final host, is always a vertebrate and becomes infected by ingesting the second intermediate host or by direct penetration of cercariae [34, 35]. Trematodes develop into adults in the definitive host and release sexually produced embryos into the environment to initiate the life cycle once again [34, 35].

At our study site in Polecat Creek, the prevalence (infection rate) of trematodes varied by native snail species and between years (Table 1). For example, in July 2015, the prevalence varied from 28.7% to 53.1% in native snails (Table 1). Yet, in 2014 and 2015, we found no trematode infections in the 427 *Potamopyrgus* that we examined (Table 1), suggesting that these non-native snails may be resistant or less competent hosts to the native trematodes occurring in Polecat Creek.

**Table 1:** Trematode infections at the field site. Trematode diversity, cercarial types, and prevalence of infection in snails from a field survey in Polecat Creek during July 2014 and 2015. The snail *Potamopyrgus antipodarum* is the only non-native snail in this ecosystem (shown in bold).

Year	Snail	Total Snails Dissected	Total Snails Infected	Prevalence	Trematode Diversity	Trematode Superfamiles ^a^
2014	*Galba*	42	4	9.5%	1	ALL
	*Physa*	80	5	6.3%	2	PRO; ALL
	*Pyrgulopsis*	226	49	21.7%	1	PRO
	***Potamopyrgus***	136	0	0.0%	0	NA
2015	*Galba*	90	33	36.7%	2	SCH; ALL
	*Physa*	122	35	28.7%	4	ECH; SCH; PRO; ALL
	*Pyrgulopsis*	49	26	53.1%	2	PRO; ALL
	***Potamopyrgus***	291	0	0.0%	0	NA

^a^ Abbreviations for the trematode Superfamilies: ALL = Allocreadiidea; ECH = Echinostomatidae; PRO = Pronocephaloidea; and SCH = Schistosomatoidae. NA indicates no infections were found in this snail.

### Experimental design

In July 2014 and 2015, we manipulated *Potamopyrgus* biomass to assess whether this non-native snail reduced prevalence of trematodes in native snails (caused a dilution effect). We created three experimental treatments: native snails only (control), native snails with the ambient biomass of non-native *Potamopyrgus*, or native snails with high biomass of non-native snails. We assessed ambient biomass of *Potamopyrgus* by collecting four quantitative Hess samples at our study site in Polecat Creek, measuring the length of all non-native *Potamogyrgus*, and calculating the mean ambient biomass using a length-mass regression [37] (2014 = 747.1 [SD +/-2.4] mg/m^2^; 2015 = 2,175.8 mg/m^2^[SD +/- 456.5]). In 2014, we used the above calculated biomass (747.1 mg/m^2^) for the ambient *Potamopyrgus* biomass treatment, but we used the ambient biomass of *Potamopyrgus* from 2009 (Krist et al., in review) (3,522 mg/m^2^ [SD +/- 3658.2]) as the high *Potamopyrgus* biomass treatment.

We collected non-native snails with hand sieves. We collected adult native *Physa* (mean 9.23 mm [SD +/- 1.0] total shell length) and *Pyrgulopsis* (2014: mean 5.85 mm [SD +/- 0.64]; 2015: mean 4.71 mm [SD +/-0.55] total shell length) from our study site at Polecat Creek. Since *Galba* is rarer and patchily distributed in Polecat Creek, in 2015, we collected *Galba* from the Snake River (into which Polecat Creek drains) at a location approximately 1.7 km from the experimental site (mean 11.73 [SD +/- 1.62] shell length). To exclude previously infected snails from our experiment, we assessed whether native snails were infected with trematodes by placing individuals in 30 mL cups under 60-watt lamps for two hours (this procedure forces the release of trematode transmission stages, cercariae, from snails). Because snails with recently acquired infections do not release parasites, we inadvertently included a small number of infected snails in our experiment. However, because these infected snails were randomly distributed among replicates, they should not have affected the outcome of the experiments. We only used adult *Potamopyrgus* (> 3.0 mm total shell length) in our experiments.

In 2014, we placed native snails (*Physa* or *Pyrgulopsis*) alone (control) or with an ambient or high biomass of non-native *Potamopyrgus* into small (144 cm^2^) and large (289 cm^2^) experimental chambers (modified square plastic sandwich containers; with mesh windows on the tops and sides to allow clean, oxygenated water and the infective stage of trematodes into the chambers). We used two sizes of chambers to increase the likelihood of success because we were concerned that the amount of water flowing into the small chambers might not be sufficient to transport enough trematode transmission stages to detect a dilution effect. To assess the dilution effect in *Physa*, we placed 8 *Physa* in each small chamber, and 12 in each large chamber (Table 2). For *Pyrgulopsis* we placed 10 snails in each small chamber, and 15 in each large chamber. Using the high biomass of *Potamopyrgus* (3,522 mg/m^2^ [SD +/- 3658.2]) and the area of the experimental chambers, the high biomass treatment was 54.23 mg for small chambers and 84.5 mg for large chambers. Thus, small chambers had 171 *Potamopyrgus* (for the mean shell length of 3.2 mm) and large chambers had 267 snails for the ambient treatments (Table 2). We haphazardly placed four replicate chambers for each of the three biomass treatments and 2 chamber sizes of *Physa* in floating platforms (24 chambers), to allow the air breathing snails access to the surface, and *Pyrgulopsis* to the stream bottom using bricks as anchors (24 chambers). Before adding snails to the experimental chambers, we added algae-covered pebbles from the stream as a food source. Twice a week, we supplemented the food with fish food pellets (Wardley Precise Portions Goldfish Food) to reduce the likelihood of parasite-induced mortality which increases with insufficient food [38]. We monitored each experimental chamber weekly during the four-week experimental period to remove dead snails, clean screens of silt and debris, and replace algae covered pebbles.

**Table 2:** Experimental designs for 2014 and 2015. The native species are *Pyrgulopsis*, *Physa*, and *Galba*, and *Potamopyrgus* is the non-native. The number of individuals of each species in each experimental chamber are noted, and the number of replicates of each treatment are in parentheses. The differences between the experimental designs between the two years were: 1) duration of the experiment in 2014 was 28 days and the duration in 2015 was 12 days, 2) small and large chambers were used in 2014 and only small chambers in 2015, and 3) four replicates per treatment in 2014 and 7–8 in 2015.

2014			
		Treatment	
Chamber Size	Control	Ambient *Potamopyrgus* Biomass	High *Potamopyrgus* Biomass
Small	10 *Pyrgulopsis* (4)	10 *Pyrgulopsis* +36 *Potamopyrgus* (4)	10 *Pyrgulopsis* + 171 *Potamopyrgus* (4)
Large	15 *Pyrgulopsis* (4)	15 *Pyrgulopsis* + 57 *Potamopyrgus* (4)	15 *Pyrgulopsis* + 267 *Potamopyrgus* (4)
Small	8 *Physa* (4)	8 *Physa* + 36 *Potamopyrgus* (4)	8 *Physa* + 171 *Potamopyrgus* (4)
Large	12 *Physa* (4)	12 *Physa* + 57 *Potamopyrgus* (4)	12 *Physa* + 267 *Potamopyrgus* (4)
2015			
Chamber Size	Control	Ambient *Potamopyrgus* Biomass	High *Potamopyrgus* Biomass
Small	11 *Pyrgulopsis* (7)	11 *Pyrgulopsis* + 90 *Potamopyrgus* (7)	11 *Pyrgulopsis* + 180 *Potamopyrgus* (7)
Small	8 *Galba* (8)	8 *Galba* + 90 *Potamopyrgus* (8)	8 *Galba* + 180 *Potamopyrgus* (8)

At the end of the experimental period, we transported the surviving native snails (mean 42.5% of *Physa* and mean 87.0% of *Pyrgulopsis;* S2 Table) to the University of Wyoming to assess infection levels among treatments. We housed the snails in a flow through system (complete water changes four times/day) for sixteen weeks, the time required for the trematode parasites to develop to a stage that is easily detectable. To ensure that we accounted for all infections, we 1) monitored mortality three times/week and immediately dissected and measured the shell length (using calipers) of all dead snails to assess infection status, 2) biweekly we exposed all snails to light (60-watt lamps for two hours) to force trematode transmission stages (cercariae) from snails with highly developed infections, and 3) at sixteen weeks, we dissected all remaining snails to determine infection status and measured their shell length.

**Fig 1:**
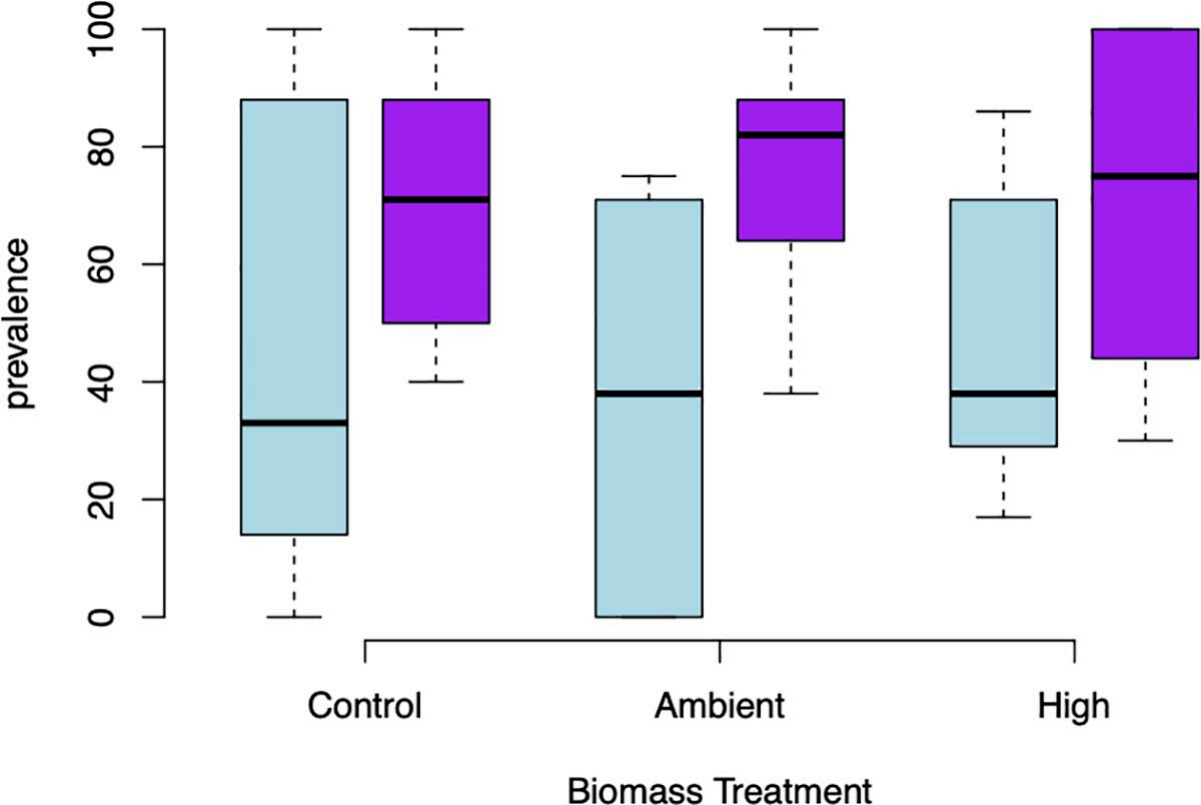
Infection status by snail taxon. Prevalence, the percentage of infected individuals per replicate, of all trematode infections by Biomass treatment (Control, with no *Potamopyrgus*, Ambient levels of *Potamopyrgus* in Polecat Creek, and High biomass) for *Galba* (light blue) and *Pyrgulopsis* (purple). Boxplots show the median, the interquartile range (shown by the upper and lower edges of the box), and the 1.5 interquartile ranges (whiskers).

Because mortality was very high in half of the *Physa* treatments (S2 Table) and prevalence was very high in all *Pyrgulopsis* treatments (Fig 1, S2 Table), we repeated the experiment in summer 2015 at the same site in Polecat Creek. To reduce mortality, we only used small chambers in the 2015 experiment because snails survived better in small relative to large chambers (S2 Table), and we used the native snail *Galba* instead of *Physa*, because of high mortality of *Physa* in 2014 (S2 Table). Because the infection rate was so high in 2014, we were concerned that we did not observe a dilution effect simply because the presence of so many trematodes swamped out any possible benefit of the non-native snail. So, to reduce the number of infections in 2015, we shortened the duration of time that snails were exposed to trematode transmission stages by over 50% (from 28 to 12 days). We also doubled the number of replicates relative to the first experiment, and, for the ambient biomass treatment, we used ambient levels of biomass of *Potamopyrgus* for 2015 in Polecat Creek and twice that amount for the high *Potamopyrgus* biomass treatment. We monitored the experimental chambers every other day to remove any dead snails and clean silt and debris from the mesh windows. We placed native snails (8 *Galba* or 11 *Pyrgulopsis*: Table 2) alone (control) or with the non-native *Potamopyrgus* (at ambient and high biomass) into experimental chambers with algae-covered rocks. Based on the ambient biomass of *Potamopyrgus* in Polecat Creek in 2015 (2,175.8 mg/m^2^ [+/- 456.5]) and the area of the experimental chambers, the ambient biomass treatment was 28.29 mg or 90 *Potamopyrgus* (for the mean shell length of 3.2 mm) and two times that amount (56.58 mg or 180 snails) for the high *Potamopyrgus* biomass treatments (Table 2). We haphazardly placed eight replicate chambers for each of the three treatments (24 chambers total) of *Galba* in floating platforms, to allow the air breathing snails access to the surface, and seven replicate chambers of *Pyrgulopsis* (21 chambers total) to the stream bottom using bricks as anchors. There were fewer replicates of *Pyrgulopsis* because of chamber failure (before we deployed chambers in the stream). As in the 2014 experiment, we supplemented food with fish food pellets biweekly, we housed all surviving snails (83.9% of *Galba* and 92.4% of *Pyrgulopsis;* S2 Table) in a flow through system for sixteen weeks at the University of Wyoming to allow time for trematode development, and we monitored mortality and assessed infections in the snails using the same methods as in 2014. The experimental design is summarized in Table 2.

In both years, small (< 2.5 mm) *Potamopyrgus* from the stream migrated into the experimental chambers altering the discrete levels of biomass that we originally manipulated for each experimental biomass treatment. Therefore, at the end of the experiments, we collected and preserved (in 95% ethanol) all *Potamopyrgus*, measured shell length with digital calipers, and used a length-mass regression [32] to assess *Potamopyrgus* biomass for each replicate. We divided the total mass by the area of the experimental chamber to obtain the biomass (mg/m^2^) of *Potamopyrgus* for each replicate (2014: range 0–71.5 mg/m^2^ with *Physa*, and range 3.9–88.7 mg/m^2^ with *Pyrgulopsis*; 2015: range 0–97.0 mg/m^2^ for *Galba*, range 0.6–77.8 mg/m^2^ with *Pyrgulopsis)*.

### Statistical analyses

We used Bayesian multilevel models using the probabilistic programming language Stan [39, 40] to assess whether the biomass of *Potamopyrgus* or size of the native snail affected infection status of native snails. We analyzed each native snail taxon separately as distinct tests of the dilution effect hypothesis. However, we were unable to analyze the dilution effect hypothesis for the native snail *Physa* because sample sizes were so small (from high mortality) that the models could not compile. For the two remaining native snail taxa, *Pyrgulopsis* and *Galba*, we used individual infection status as the dependent variable (Bernoulli distribution). We could not use trematode prevalence because the sample sizes of surviving native snails/replicate were insufficient to calculate accurate prevalence rates (e.g. for the 2015 experiment: 4–8 individuals/chamber for *Galba* and 7–11 individuals/chamber for *Pyrgulopsis*). We analyzed individual infection status in two ways: combining all types of trematodes and with specific trematode taxa (SuperFamily) because differences in host specificity and infection mode suggest trematode taxa may not be uniformly reduced by the presence of a single less competent host (*Potamopyrgus*). We had sufficient sample sizes to analyze two SuperFamilies that infected *Pyrgulopsis*: Pronocepahaloidea (n = 163) and Allocreadiidea, (n = 104). There were not enough infections of a single trematode taxon to assess specific trematode taxa in *Galba*.

For all models, the population level (fixed) effects were biomass of *Potamopyrgus* and size (shell length) of the native snail (because larger snails are often infected at higher rates by trematodes than smaller individuals, [e.g. 41–43]. Because small snails migrated into the experimental chambers, we ran models with both the original biomass treatments (excluding the migrants) and the total biomass of *Potamopyrgus* in each chamber (including the migrants) to learn whether the migrant snails altered the likelihood of detecting a dilution effect. Because the interaction between *Potamopyrgus* biomass and size of the native snail was not significant in any of the models, we omitted this interaction term from the final models. In the analysis of biomass treatment excluding the migrants (Tables 3 and 4) the group level effect (also called random effect) was replicate (nested in treatment), and for *Pyrgulopsis* only, year that the experiment occurred and size of the experimental chamber size were also included as group level effects (*Galba* was only studied in one year, 2015, and with one size of experimental chamber). For the analysis of total biomass of *Potamopyrgus* including the migrants (S3 and S4 Tables) the group level effects for *Pyrgulopsis* were year that the experiment occurred and size of the experimental chamber size. There were no group level effects in the models for *Galba*. We used program R with the brms package [39] to compile the Bayesian multilevel effect models. To evaluate whether the models converged, we visually inspected the traceplots to see that the chains mixed well, and only used models with Rhat equal to 1, a metric revealing that the models converged.

**Table 3:** Model coefficients and credible intervals for overall infection status. How infection status (all trematodes combined) was affected by biomass of *Potamopyrgus* (Biomass Treatment: Control, with no *Potamopyrgus*, ambient levels of *Potamopyrgus* in Polecat Creek, and High biomass) for two taxa of native snails in Bayesian multilevel models [39, 40]. For the native snail *Pyrgulopsis* (A*)*, the group level effects were the year that the experiment was conducted (year), the size of the experimental chamber (chamber size) and the experimental replicate (replicate) nested within Treatment. Because we only tested the dilution effect hypothesis with the native snail *Galba* (B) in one year and with all the same sized experimental chambers, the group level effect for *Galba* was replicate. Variables that significantly affected infection status possess 95% credible intervals that exclude zero and are bolded. For the group level effects, values in the Coefficients column are standard deviations for the Coefficient estimates.

**A**	*Pyrgulopsis*	Effect level	Coefficients	95% Credible Interval
	**Intercept**	**population**	**-4.90**	**-8.61 –-1.57**
	Biomass Treatment	population	0.07	-0.30–0.44
	**Size of native snail**	**population**	**1.09**	**0.65–1.56**
	**Chamber Size**	**group**	**1.15**	**0.04–4.53**
	**Year**	**group**	**1.33**	**0.06–4.72**
	**Replicate**	**group**	**0.25**	**0.01–0.70**
**B**	*Galba*	Effect Type	Coefficients	95% Credible Interval
	**Intercept**	**population**	**-4.30**	**-7.49 –-1.32**
	Biomass Treatment	population	0.05	-0.71–0.81
	**Size of native snail**	**population**	**0.33**	**0.11–0.59**
	**Replicate**	**group**	**0.75**	**0.08–1.84**

**Table 4:** Model coefficients and credible intervals for specific trematode taxa. How infection status of the native snail *Pyrgulopsis* by trematodes of the SuperFamilies (A) Pronocephaloidea and (B) Allocreadiidea was affected by biomass of *Potamopyrgus* (Biomass Treatment: Control, with no *Potamopyrgus*, Ambient levels of *Potamopyrgus* in Polecat Creek, and High biomass) in Bayesian multilevel models [39, 40]. Variables that significantly affected infection status are bolded. For the group level effects, values in the Coefficients column are standard deviations for the Coefficient estimates.

**A**	Pronocephaloidea	Effect level	Coefficients	95% Credible Interval
	**Intercept**	**population**	**-5.96**	**-9.21 –-2.79**
	Biomass Treatment	population	0.22	-0.07–0.51
	**Size of native snail**	**population**	**1.01**	**0.65–1.39**
	**Chamber Size**	**group**	**1.04**	**0.03–4.07**
	**Year**	**group**	**1.19**	**0.05–4.45**
	**Replicate**	**group**	**0.14**	**0.01–0.46**
**B**	Allocreadiidea	Effect Type	Coefficients	95% Credible Interval
	Intercept	population	0.28	-2.92–4.04
	Biomass Treatment	population	-0.12	-0.49–0.29
	Size of native snail	population	-0.19	-0.58–0.19
	**Chamber Size**	**group**	**0.98**	**0.02–4.08**
	**Year**	**group**	**1.66**	**0.29–5.04**
	**Replicate**	**group**	**0.28**	**0.01–0.81**

## Results

**Fig 2:**
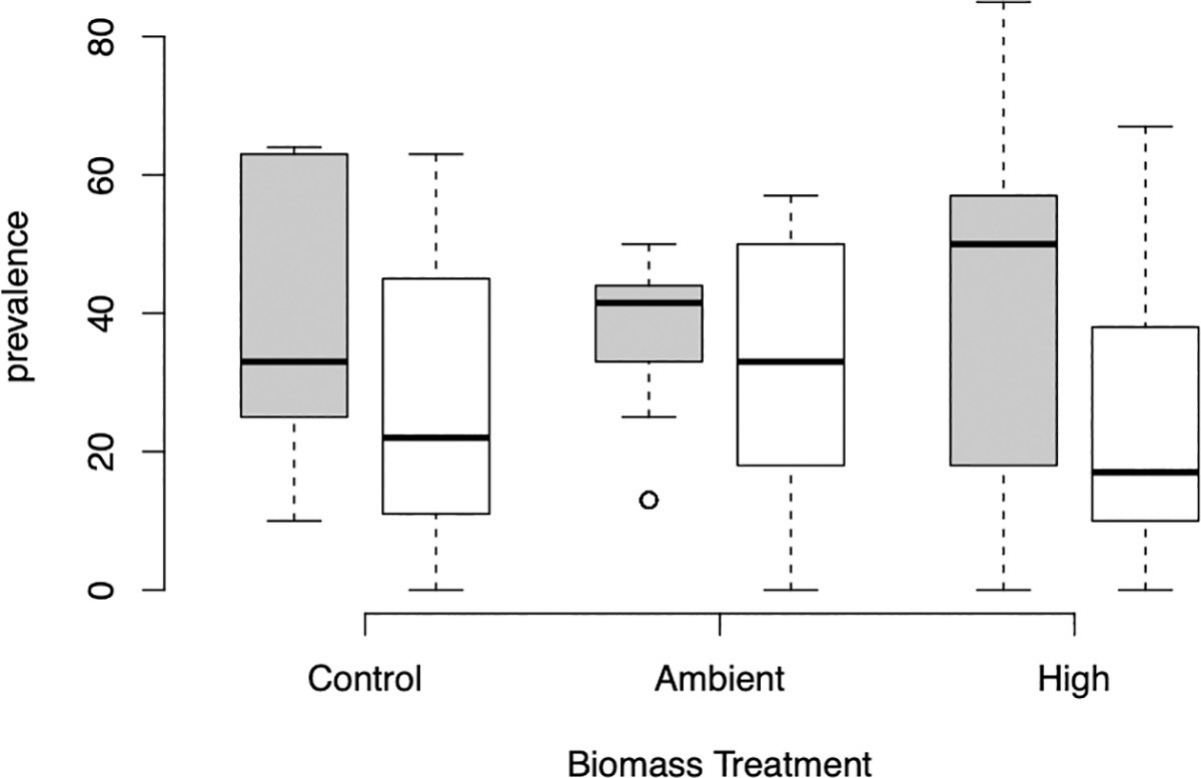
Infection status in *Pyrgulopsis* by two most common trematode taxa. Prevalence, the percentage of infected individuals per replicate, of trematode infections in the native snail *Pyrgulopsis* by Biomass treatment (Control, with no *Potamopyrgus*, ambient levels of *Potamopyrgus* in Polecat Creek, and High biomass), for the Superfamilies Pronocephaloidea (gray), and Allocreadiidea (white). Boxplots show the median, the interquartile range (shown by the upper and lower edges of the box), and the 1.5 interquartile ranges (whiskers). Extreme values are shown in open circles.

**Fig 3:**
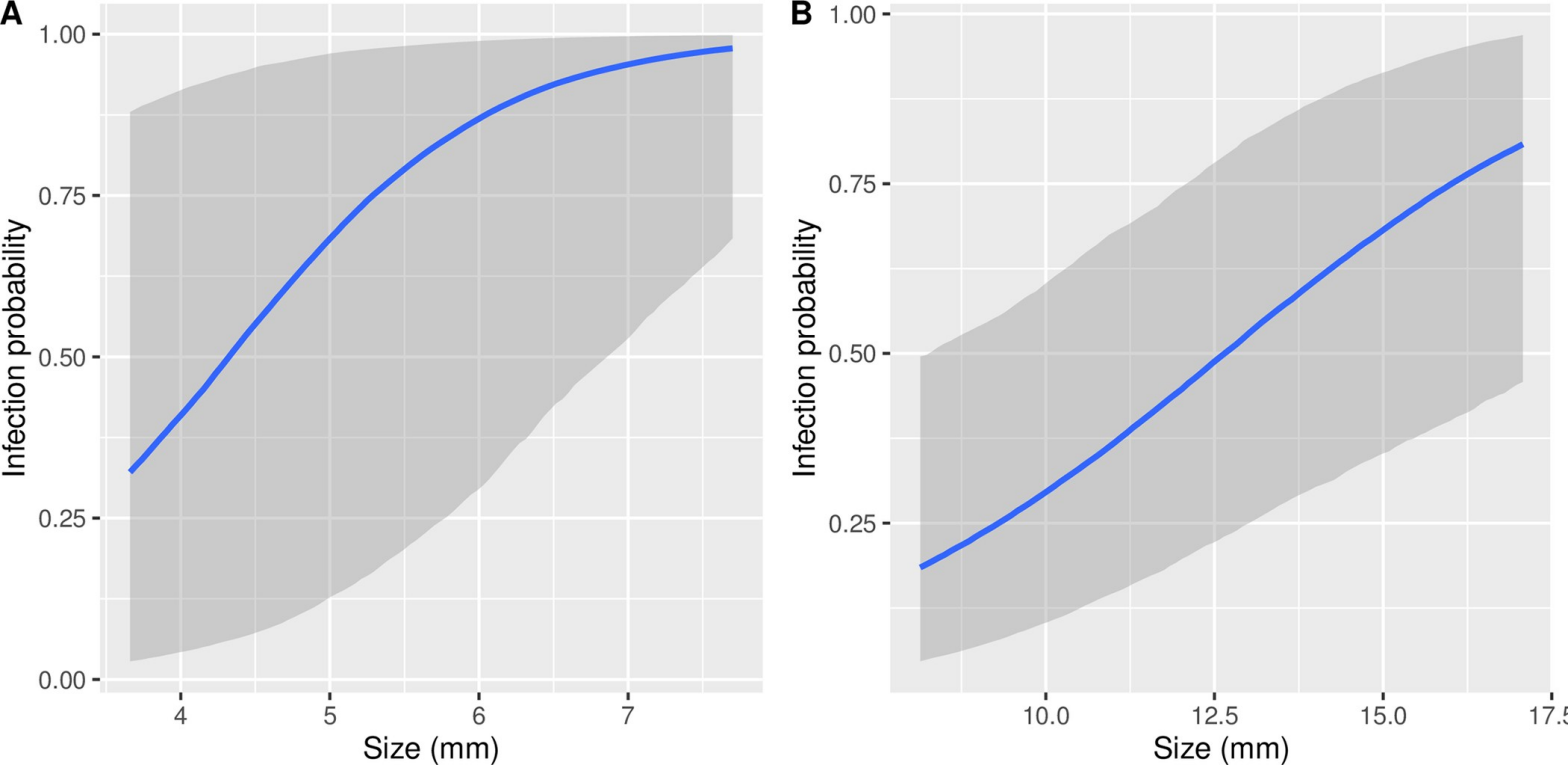
Native snail length and infection status. The predicted effects, with 95% credible intervals, of shell length of the native snails a) *Pyrgulopsis* and b) *Galba* on infection status, of all trematodes combined, from the models in Table 3. Infection rates were higher in *Pyrgulopsis* and increased more steeply with shell length until most *Pyrgulopsis* > 6 mm were infected by trematodes.

We found no evidence for a dilution effect; the presence or level of biomass of the non-native *Potamopyrgus* did not reduce overall infection status in either of the native snails (Fig 1, Table 3, S3 Table). Infection status by the two most common trematodes infecting *Pyrgulopsis* were also not affected by the presence or biomass of *Potamopyrgus* (Fig 2, Table 4, S4 Table). Infection levels increased with size of the native snail for all trematodes combined (Fig 3, Table 3, S3 Table). Relative to *Galba*, infection levels were higher in *Pyrguplopsis* and increased more steeply with size until nearly all *Pyrgulopsis* > 6 mm were infected with trematodes. Infection levels also increased for one of the two most common trematodes (Super Family Pronocephaloidea; Table 4, S4 Table).

## Discussion

We found no evidence for the dilution effect hypothesis in the two native snails we examined. The presence of an invasive species can reduce infection levels in a native species directly by serving as a target for, consuming, or physically or chemically obstructing the parasite [4, 6–12], or indirectly by affecting the native host’s abundance via competition, or by affecting the native host’s behavior or physiological condition [5, 16, 19]. In our controlled experiments, the abundance of the native host was held constant, and because the snails were limited in space within the chambers, changes in behavior of the native species due to interactions with the non-native were unlikely to affect the probability of encountering a parasite transmission stage (miracidium). Thus indirect means of influencing infection were unlikely to have occurred in our experiment. Although we cannot rule out the possibility that reduced biodiversity in the chambers altered the physiological condition of either the non-native or the native snails thereby changing infection rates, we noticed no visible changes in the condition or behavior of any of the snails used in the experiments. Also, we did not detect differences in the infection rates in the native species among treatments suggesting that the presence of *Potamopyrgus* did not alter the condition, physiology, or behavior of the natives in ways that influenced transmission. Therefore, if we had found evidence for a dilution effect, it would most likely have been caused by *Potamopyrgus* being a less competent target host. Since we did not detect a dilution effect, *Potamopyrgus* is probably not an effective “decoy” for the trematodes that infect these native species in the study stream.

Our results are inconsistent with many studies showing that non-native animals from brown trout [22] and bumble bees [23] to bank voles [21] reduce infection rates in native animals. Our results also contrast with a recent meta-analysis of dilution effect experiments showing that increased host diversity decreased the abundance of parasites across a wide range of parasite types, life cycles, and functional groups [16]. However, in the meta-analysis only about a half (53%) of the studies addressed the addition of one host species, like in our experiments, and the dilution effect appeared stronger when single-species addition studies were removed. Thus, perhaps the evidence for the dilution effect is not as strong in this subset of the studies.

A probable reason that we did not find a dilution effect is that miracidia (trematode transmission stage) are very host specific and thus did not attempt to infect *Potamopyrgus*. Most trematodes have evolved highly specific relationships to their snail hosts (genus or family) [44]. For example, in a pond population of the snail *Helisoma anceps*, seven of the eight trematode taxa infecting *H*. *anceps* only infected that snail species [42]. In extreme cases, specific strains of trematodes can only infect one phenotype of a snail species (compatibility polymorphism) [45] and specificity can even vary among host populations [46]. However, other studies of trematodes and gastropods have found that unintended snail hosts can be infected by trematode parasites (cause a dilution effect). For example, non-host snails can become infected with trematodes that typically infect the intended host species, but variation among infection rates of non-host snails suggests attractiveness or susceptibility to the parasites is not uniform among non-host snails [47–49]. Infection rates of *Schistosoma mansoni* in *Biomphalaria glabrata* snails (the intended host) were reduced when non-susceptible snails were also present [47, 50]. Although, we found a few infected *Potamopyrgus* in Polecat Creek in 2017 [31], revealing that miracidia of one trematode taxon (Superfamily Echinostomatidae) can infect the non-native snail, Echinostomatidae infections were rare or absent in the two native snails we analyzed (0.2% in *Pyrgulopsis*, 0% in *Galba*) suggesting that susceptibility of *Potamopyrgus* to this one trematode taxon would have little, if any, effect on infection levels in these native snails. In contrast, Echinostomatidae infections were common in the native snail *Physa* (33%) suggesting that *Potamopygus* could possibly dilute those trematodes in *Physa*. However, we did not have adequate sample sizes to test this possibility because of high *Physa* mortality in the experiment. Thus, while host specificity likely does not explain why all trematode taxa failed to infect the invasive snail, it probably explains why most trematodes could not infect *Potamopyrgus*.

A dilution effect can be caused by the presence of alternative hosts serving as targets for parasites, or by concomitant altered diversity or changes in relative species abundance [16, 51]. In our experiment we added a single species that could serve as an unintended target, and thus also increased species diversity in the experimental chambers. If we had found a dilution effect, it could have been caused by either altered species diversity, relative abundance, or *Potamopyrgus* acting as a less effective target. Because we did not vary the abundance or density of the native species, it remains possible that in some populations *Potamopyrgus* could cause a dilution effect indirectly by competing with native species. Also, our experiments were not designed to and did not effectively address how altering biodiversity could result in a dilution effect. Thus, indirect mechanisms of the dilution effect remain a possibility in this ecosystem.

In our experiments, larger snails were more likely to be infected by all trematode parasites combined and by members of the SuperFamily Pronocephaloidea (Tables 3 and 4). This finding is consistent with many studies where snails infected with trematodes are larger than uninfected snails [e.g. 41–43, 52]. Possible causes for this relationship include trematodes inducing higher growth rates of host snails (gigantism; [52–54]), larger snails having longer exposure because they are older (exposure duration), and differences in susceptibility between larger, adult snails and smaller, juvenile snails to some trematode taxa. In our experiment, all of these are possible explanations for why larger snails were more likely to be infected. Gigantism could explain why infected snails were larger than uninfected snails because we measured snail size at the end of the experiment after most snails had been exposed to infection for 18–20 weeks. Given that many experiments document growth rates of snails over a few weeks, the duration of infection was probably long enough to detect differences in growth rates between infected and uninfected snails [52] if they occurred. Our results could also be explained by duration of exposure; larger snails are older and are likely to be infected with trematodes simply because they are exposed for a longer period of time than younger, smaller snails. Even though we omitted all snails with transmissible (patent) infections from the experiment, high prevalence of trematodes at the study site means that inevitably we included some snails in the experiments that were already harboring early, undetectable infections. Finally, it is also possible that larger, adult snails are more susceptible than juveniles to some of the trematode taxa in Polecat Creek. We found that infection frequency increased with snail size in trematodes in the Superfamily Pronocephaloidea and not in the Allocreadiidea. Juvenile snails can be less [e.g. 55] or more [e.g. 42] susceptible to specific trematode taxa than adults.

For the group-level effects (also called random effects), infection status consistently varied among replicates and chamber size and infection rates were higher in 2014. Variation among replicates probably resulted by chance: the sample sizes of native snails in each replicate were sufficiently small (Table 2) that minor differences in the number of infected individuals had large effects on the infection status of single replicates. Infection rates were higher in large chambers than in small chambers, probably because larger chambers permitted a higher volume of water to flow through them, thereby increasing the frequency that water-borne trematode transmission stage (miracidia) entered the chambers. For *Pyrgulopsis*, infection status was also higher in 2014 than in 2015 because the duration of exposure to infection was over twice as long in 2015 (Table 4).

In conclusion, we found no evidence that the non-native snail, *Potamopyrgus*, reduces infection rates (produces a dilution effect) in the native snails that we examined. Perhaps our results are not uncommon because negative results are less likely to be published. However, our inability to detect a dilution effect highlights the importance of investigating the underlying mechanisms that are required for a dilution effect to occur and the factors that reduce or prevent it. Consistent with many previous studies, our results suggest that community composition is crucial to creating a dilution effect [e.g. 16]. In our case, *Potamopyrgus* does not appear to be a suitable target for most of the trematodes in this community and thus does not sufficiently serve as a decoy for parasites of the native species. A better understanding of the mechanisms required for the dilution effect to occur will improve our understanding of parasite-host interactions and dynamics, which is crucial to predicting how multiple anthropogenic alterations will affect disease occurrence and prevalence. In addition, when invasive species fail to cause dilution effects in native species that they compete with, the lack of the dilution effect could contribute to the invasive species success by releasing the invasive species from infection by the same parasites that lower fitness of the competing native species.

## Supporting information

S1 Table Ambient snail densities (individuals/m^2^) from Polecat Creek. Over a 17-year period, the distribution of each of the species can be very patchy and varies within and between years.
(DOCX)

S2 Table Native snail survival and prevalence. We found that size of the experimental chamber greatly affected survival of some native snails in our 2014 experiment. Thus, in 2015 we used only small chambers because snails survived in them at higher rates in the 2014 experiment. We report the snails following exposure to trematode parasites, for each treatment and by chamber size, in 2014 and 2015. We also show the biomass of *Potamopyrgus* (non-native) at the beginning and the end of the experiment and the infection rate (mean prevalence) in the native snails at the end of the experiment.
(DOCX)

S3 Table Infection status including *Potampyrgus* migrants. Model coefficients and credible intervals describing how infection status (all trematodes combined) was affected by biomass of *Potamopyrgus* (including unintended *Potampyrgus* migrants) for two taxa of native snails in Bayesian multilevel models [41–42]. For the native snail *Pyrgulopsis* (A), the group level effects were the year that the experiment was conducted (year) and the size of the experimental chamber (chamber size). Because we only tested the dilution effect hypothesis with the native snail *Galba* (B) in one year and with all the same sized experimental chambers, there were no group level effects for *Galba*. Variables that significantly affected infection status possess 95% Credible intervals that exclude zero and are bolded. For the group level effects, values in the Coefficients column are standard deviations for the Coefficient estimates.
(DOCX)

S4 Table Specific trematode taxa including *Potampyrgus* migrants. Model coefficients and credible intervals describing how infection status of the native snail *Pyrgulopsis* by trematodes of the SuperFamilies (A) Pronocephaloidea and (B) Allocreadiidea were affected by biomass of *Potamopyrgus* (including unintended *Potampyrgus* migrants) in bayesian multilevel models [41, 42]. Variables that significantly affect infection status are bolded. Group level effects were size of the experimental chambers and year. For the group level effects, values in the Coefficients column are standard deviations for the Coefficient estimates.
(DOCX)

S1 Data *Pyrgulopsis* data. (CSV)

S2 Data *Galba* data. (CSV)

S3 Data brms R code and output. (DOCX)
