# Supplementary material for: No evidence for a dilution effect of the non-native snail, Potamopyrgus antipodarum, on native snails
Source: PLoS One. 2020 Oct 1;15(10):e0239762. doi: 10.1371/journal.pone.0239762 (PMC7529281; doi:10.1371/journal.pone.0239762)
Supplement: S1 Table — Over a 17-year period, the distribution of each of the species can be very patchy and varies within and between years. (DOCX) [file pone.0239762.s001.docx]

**Supplemental Table 1. Ambient snail densities (individuals/m^2^) from Polecat Creek.**

Over a 17-year period, the distribution of each of the species can be very patchy and varies within and between years.

|  | *Potamopyrgus* | *Pyrgulopsis* | *Physa* | *Galba* |
| --- | --- | --- | --- | --- |
| 2000-2001^1^ | 254,098 | 0 | 6 | 79 |
|  |  |  |  |  |
| 2009^2^ | 39,809.1 | 13.7 | 22.8 | 252.7 |
|  |  |  |  |  |
| 2014^3^ |  |  |  |  |
| Site 1 | 2355.9 | 6.2 | 80.2 | 0 |
| Site 2 | 968.3 | 0 | 0 | 0 |
| Site 3 | 1036.1 | 1085.4 | 0 | 0 |
|  |  |  |  |  |
| 2015^3^ |  |  |  |  |
| Site 1 | 2198.6 | 3.1 | 30.8 | 0 |
| Site 2 | 407.0 | 30.8 | 89.4 | 0 |
| Site 3 | 1443.1 | 1218.0 | 30.8 | 0 |
|  |  |  |  |  |
| 2016^4^ | 36,928.2 | 16.7 | 66.2 | 41.7 |

^1^Data from Hall et al. [32], showing average annual density averaged over 12 months.

^2^Data from Krist et al. (in review) averaged across six samples/month in June, July, August and September 2009.

^3^Data from Larson and Krist [34] from three different sites within 200 m of the location of the present experiment.

^4^Data from Greenwood, DJ, Hall RO, Tibbets TM, Krist AC. A precipitous decline in an invasive snail cannot be explained by a native predator. Biol Invasions. 2020;22: 363-378 from one site near the location of the present study showing average densities over four months (June-September).
