# Supplementary material for: No evidence for a dilution effect of the non-native snail, Potamopyrgus antipodarum, on native snails
Source: PLoS One. 2020 Oct 1;15(10):e0239762. doi: 10.1371/journal.pone.0239762 (PMC7529281; doi:10.1371/journal.pone.0239762)
Supplement: S2 Table — We found that size of the experimental chamber greatly affected survival of some native snails in our 2014 experiment. Thus, in 2015 we used only small chambers because snails survived in them at higher rates in the 2014 experiment. We report the snails following exposure to trematode parasites, for each treatment and by chamber size, in 2014 and 2015. We also show the biomass of Potamopyrgus (non-native) at the beginning and the end of the experiment and the infection rate (mean prevalence) in the native snails at the end of the experiment. (DOCX) [file pone.0239762.s002.docx]

**Supplemental Table 2.** **Experimental Details.**

Because snails survived in small chambers at higher rates than in large chambers in 2014, in 2015 we used only small chambers. For 2014 and 2015, we report the percent survival of three native snails following exposure to trematode parasites, by chamber size, and by treatment. We also show the mean biomass of *Potamopyrgus* (non-native) at the beginning and the end of the experiment and the mean infection rate (prevalence) in the native snails at the end of the experiment.

| **2014** |  |  |  |  |  |  |  |  |
| --- | --- | --- | --- | --- | --- | --- | --- | --- |
| Native snail | Chamber Size | Treatment | Mean Number of Surviving Native Snails per Chamber | Mean Initial Number of Native Snails per Chamber | Percent Survival | Initial *Potamopyrgu*s biomass (mg) | Mean ending *Potamopyrgu*s biomass (mg) | Mean Parasite Prevalence in Native Snails (% infected) |
| *Physa* sp. | Small | Native only | 5.25 | 8 | 65.6 | 0 | 0.08 | 44.0 |
|  | Small | Ambient *Potamopyrgus* biomass | 6 | 8 | 75.0 | 11.5 | 8.98 | 53.8 |
|  | Small | High *Potamopyrgu*s biomass | 6.75 | 8 | 84.4 | 17.9 | 41.6 | 39.3 |
|  | Large | Native only | 2.25 | 12 | 18.8 | 0 | 0.01 | 37.5 |
|  | Large | Ambient *Potamopyrgu*s biomass | 3 | 12 | 25.0 | 54.2 | 11.6 | 58.3 |
|  | Large | High *Potamopyrgu*s biomass | 2.5 | 12 | 20.8 | 84.5 | 62.4 | 33.3 |
|  |  |  |  |  |  |  |  |  |
| *Pyrgulopsis* | Small | Native only | 10 | 10 | 100 | 0 | 20.1 | 85.8 |
|  | Small | Ambient *Potamopyrgu*s biomass | 8.75 | 10 | 87.5 | 11.5 | 17.2 | 89.2 |
|  | Small | High *Potamopyrgu*s biomass | 7.5 | 10 | 75.0 | 17.9 | 52.1 | 68.1 |
|  | Large | Native only | 12.75 | 15 | 85.0 | 0 | 6.9 | 85.1 |
|  | Large | Ambient *Potamopyrgu*s biomass | 13 | 15 | 86.7 | 54.2 | 21.8 | 88.8 |
|  | Large | High *Potamopyrgu*s biomass | 13 | 15 | 86.7 | 84.5 | 72.3 | 93.8 |
|  |  |  |  |  |  |  |  |  |
| **2015** |  |  |  |  |  |  |  |  |
| *Galba* | Small | Native only | 6.75 | 8 | 84.3 | 0 | 4.1 | 33.8 |
|  | Small | Ambient *Potamopyrgus* biomass | 6.75 | 8 | 84.3 | 28.3 | 41.6 | 34.8 |
|  | Small | High *Potamopyrgus* biomass | 6.62 | 8 | 82.8 | 56.7 | 70.1 | 47.3 |
|  |  |  |  |  |  |  |  |  |
| *Pyrgulopsis* | Small | Native only | 9.4 | 12 | 78.5 | 0 | 4.3 | 51.7 |
|  | Small | Ambient *Potamopyrgus* biomass | 9.4 | 12 | 78.5 | 28.3 | 39.7 | 53.6 |
|  | Small | High *Potamopyrgus* biomass | 8.9 | 12 | 73.8 | 56.7 | 61.8 | 51.5 |
