# Supplementary material for: No evidence for a dilution effect of the non-native snail, Potamopyrgus antipodarum, on native snails
Source: PLoS One. 2020 Oct 1;15(10):e0239762. doi: 10.1371/journal.pone.0239762 (PMC7529281; doi:10.1371/journal.pone.0239762)
Supplement: S3 Table — Model coefficients and credible intervals describing how infection status (all trematodes combined) was affected by biomass of Potamopyrgus (including unintended Potampyrgus migrants) for two taxa of native snails in Bayesian multilevel models [41–42]. For the native snail Pyrgulopsis (A), the group level effects were the year that the experiment was conducted (year) and the size of the experimental chamber (chamber size). Because we only tested the dilution effect hypothesis with the native snail Galba (B) in one year and with all the same sized experimental chambers, there were no group level effects for Galba. Variables that significantly affected infection status possess 95% Credible intervals that exclude zero and are bolded. For the group level effects, values in the Coefficients column are standard deviations for the Coefficient estimates. (DOCX) [file pone.0239762.s003.docx]

**Supplemental Table 3. Model coefficients and credible intervals for all trematodes combined.**

How infection status (all trematodes combined) was affected by biomass of *Potamopyrgus* (including unintended *Potampyrgus* migrants) for two taxa of native snails in Bayesian multilevel models (brms; Bruckner 2017). For the native snail *Pyrgulopsis* (A*)*, the group level effects were the year that the experiment was conducted (year) and the size of the experimental chamber (chamber size). Because we only tested the dilution effect hypothesis with the native snail *Galba* (B) in one year and with all the same sized experimental chambers, there were no group level effects for *Galba*. Variables that significantly affected Infection status possess 95% Credible intervals that exclude zero and are bolded. For the group effects, values in the Coefficients column are standard deviations for the Coefficient estimates.

| A | *Pyrgulopsis* | Effect level | Coefficients | 95% Credible Interval |
| --- | --- | --- | --- | --- |
|  | Intercept | population | -4.92 | 1.78 – -8.59 |
|  | *Potamopyrgus* Biomass | population | 0.01 | -0.00 – 0.02 |
|  | **Size of native snail** | **population** | **1.07** | **0.23 – 0.63** |
|  | **Chamber Size** | **group** | **1.10** | **0.04 – 4.40** |
|  | **Year** | **group** | **1.34** | **0.07-4.78** |
|  |  |  |  |  |
| B | *Galba* | Effect Type | Coefficients | 95% Credible Interval |
|  | **Intercept** | **population** | **-3.43** | **-6.05 – -0.99** |
|  | *Potamopyrgus* Biomass | population | 0.00 | -0.01 – 0.01 |
|  | **Size of native snail** | **population** | **0.28** | **0.08 – 0.50** |
