# Supplementary material for: No evidence for a dilution effect of the non-native snail, Potamopyrgus antipodarum, on native snails
Source: PLoS One. 2020 Oct 1;15(10):e0239762. doi: 10.1371/journal.pone.0239762 (PMC7529281; doi:10.1371/journal.pone.0239762)
Supplement: S4 Table — Model coefficients and credible intervals describing how infection status of the native snail Pyrgulopsis by trematodes of the SuperFamilies (A) Pronocephaloidea and (B) Allocreadiidea were affected by biomass of Potamopyrgus (including unintended Potampyrgus migrants) in bayesian multilevel models [41, 42]. Variables that significantly affect infection status are bolded. Group level effects were size of the experimental chambers and year. For the group level effects, values in the Coefficients column are standard deviations for the Coefficient estimates. (DOCX) [file pone.0239762.s004.docx]

**Supplemental Table 4. Model coefficients and credible intervals for specific trematode taxa.** How infection status of the native snail *Pyrgulopsis* by trematodes of the SuperFamilies (A) Pronocephaloidea and (B) Allocreadiidea were affected by biomass of *Potamopyrgus* (including unintended *Potampyrgus* migrants) in bayesian multilevel models (brms; Brückner 2017). Variables that significantly affect infection status are bolded. For the group level effects, values in the Coefficients column are standard deviations for the Coefficient estimates.

| A | Pronocephaloidea | Effect level | Coefficients | 95% Credible Interval |
| --- | --- | --- | --- | --- |
|  | **Intercept** | **population** | **-5.96** | **-8.87 – -2.94** |
|  | *Potamopyrgus* Biomass | population | 0.01 | 0.00 – 0.02 |
|  | **Size of native snail** | **population** | **1.01** | **0.65 – 1.38** |
|  | **Chamber Size** | **group** | **0.98** | **0.02 – 3.92** |
|  | **Year** | **group** | **1.16** | **0.04-4.38** |
| B | Allocreadiidea | Effect Type | Coefficients | 95% Credible Interval |
|  | Intercept | population | 0.13 | -3.06 – 3.55 |
|  | *Potamopyrgus* Biomass | population | 0.00 | -0.01 – 0.01 |
|  | Size of native snail | population | -0.19 | -0.58 – 0.17 |
|  | **Chamber Size** | **group** | **0.99** | **0.03 – 4.13** |
|  | **Year** | **group** | **1.71** | **0.27 – 4.52** |
