# Supplementary material for: No evidence for a dilution effect of the non-native snail, Potamopyrgus antipodarum, on native snails
Source: PLoS One. 2020 Oct 1;15(10):e0239762. doi: 10.1371/journal.pone.0239762 (PMC7529281; doi:10.1371/journal.pone.0239762)
Supplement: S3 Data — (DOCX) [file pone.0239762.s007.docx]

BRMS analyses for Larson et al. “No evidence for a dilution effect of the non-native snail, *Potamopyrgus antipodarum*, on native snails”

**Testing dilution effect in the native snail, *Pyrgulopsis*:**

library(brms)

Pyrg <- read.csv("Pyrg.csv", header = TRUE)

**Analysis of Pyrgulopsis without *Potamopyrgus* migrants:**

PyrgNoMigrants <- brm(InfectionStatus ~ (1|Rep)+(0+Treatment|Rep) + (1|CageSize) + (1|year) + Treatment + Size, data=Pyrg, family="bernoulli", control = list(adapt_delta=0.99))

#no errors! Examine plots:

plot(PyrgNoMigrants)

summary(PyrgNoMigrants)

# Family: bernoulli

# Links: mu = logit

# Formula: InfectionStatus ~ (1 | Rep) + (0 + Treatment | Rep) + (1 | CageSize) + (1 | year) + Treatment + Size

# Data: Pyrg (Number of observations: 417)

# Samples: 4 chains, each with iter = 2000; warmup = 1000; thin = 1;

# total post-warmup samples = 4000

#

# Group-Level Effects:

# ~CageSize (Number of levels: 2)

# Estimate Est.Error l-95% CI u-95% CI Rhat Bulk_ESS Tail_ESS

# sd(Intercept) 1.15 1.19 0.04 4.53 1.00 1960 2082

#

# ~Rep (Number of levels: 8)

# Estimate Est.Error l-95% CI u-95% CI Rhat Bulk_ESS Tail_ESS

# sd(Intercept) 0.26 0.21 0.01 0.77 1.00 1332 1425

# sd(Treatment) 0.25 0.19 0.01 0.70 1.00 1298 1638

#

# ~year (Number of levels: 2)

# Estimate Est.Error l-95% CI u-95% CI Rhat Bulk_ESS Tail_ESS

# sd(Intercept) 1.33 1.31 0.06 4.72 1.00 1488 1395

#

# Population-Level Effects:

# Estimate Est.Error l-95% CI u-95% CI Rhat Bulk_ESS Tail_ESS

# Intercept -4.90 1.75 -8.61 -1.57 1.00 2409 2436

# Treatment 0.07 0.19 -0.30 0.44 1.00 2924 2598

# Size 1.09 0.23 0.65 1.56 1.00 3612 3248

#

# Samples were drawn using sampling(NUTS). For each parameter, Bulk_ESS

# and Tail_ESS are effective sample size measures, and Rhat is the potential

# scale reduction factor on split chains (at convergence, Rhat = 1).

**Analysis of Pyrgulopsis with migrants:**

PyrgMigrants <- brm(InfectionStatus ~ (1|CageSize) + (1|year) + NZMSBiomassMigrants + Size, data=Pyrg, family="bernoulli", control = list(adapt_delta=0.99))

#no errors

plot(PyrgMigrants)

#look good

summary(PyrgMigrants)

# Family: bernoulli

# Links: mu = logit

# Formula: InfectionStatus ~ (1 | CageSize) + (1 | year) + NZMSBiomassMigrants + Size

# Data: Pyrg (Number of observations: 417)

# Samples: 4 chains, each with iter = 2000; warmup = 1000; thin = 1;

# total post-warmup samples = 4000

#

# Group-Level Effects:

# ~CageSize (Number of levels: 2)

# Estimate Est.Error l-95% CI u-95% CI Rhat Bulk_ESS Tail_ESS

# sd(Intercept) 1.10 1.17 0.04 4.40 1.00 1629 1941

#

# ~year (Number of levels: 2)

# Estimate Est.Error l-95% CI u-95% CI Rhat Bulk_ESS Tail_ESS

# sd(Intercept) 1.34 1.31 0.07 4.78 1.00 1077 1049

#

# Population-Level Effects:

# Estimate Est.Error l-95% CI u-95% CI Rhat Bulk_ESS Tail_ESS

# Intercept -4.92 1.78 -8.59 -1.41 1.00 1802 2071

# NZMSBiomassMigrants 0.01 0.00 -0.00 0.02 1.00 4248 2686

# Size 1.07 0.23 0.63 1.54 1.00 2598 2838

#

# Samples were drawn using sampling(NUTS). For each parameter, Bulk_ESS

# and Tail_ESS are effective sample size measures, and Rhat is the potential

# scale reduction factor on split chains (at convergence, Rhat = 1).

**Testing dilution effect in the native snail, *Galba*:**

**Analysis of *Galba* without migrants:**

GalbaNoMigrants <- brm(InfectionStatus ~ (1|Rep) + (0 + Treatment|Rep) + Treatment + Size, data=Galba, family="bernoulli")

#no errors!

plot(GalbaNoMigrants)

summary(GalbaNoMigrants)

# Family: bernoulli

# Links: mu = logit

# Formula: InfectionStatus ~ (1 | Rep) + (0 + Treatment | Rep) + Treatment + Size

# Data: Galba (Number of observations: 161)

# Samples: 4 chains, each with iter = 2000; warmup = 1000; thin = 1;

# total post-warmup samples = 4000

#

# Group-Level Effects:

# ~Rep (Number of levels: 8)

# Estimate Est.Error l-95% CI u-95% CI Rhat Bulk_ESS Tail_ESS

# sd(Intercept) 1.45 0.60 0.57 2.84 1.01 1026 2092

# sd(Treatment) 0.75 0.45 0.08 1.84 1.00 912 1288

#

# Population-Level Effects:

# Estimate Est.Error l-95% CI u-95% CI Rhat Bulk_ESS Tail_ESS

# Intercept -4.30 1.57 -7.49 -1.32 1.00 3633 2933

# Treatment 0.05 0.38 -0.71 0.81 1.00 2087 1784

# Size 0.33 0.12 0.11 0.59 1.00 4301 3074

#

# Samples were drawn using sampling(NUTS). For each parameter, Bulk_ESS

# and Tail_ESS are effective sample size measures, and Rhat is the potential

# scale reduction factor on split chains (at convergence, Rhat = 1).

**Analysis of *Galba* with migrants:**

GalbaMigrants <- brm(InfectionStatus ~ NZMSBiomassMigrants + Size, data=Galba, family="bernoulli")

#no errors!

plot(GalbaMigrants)

summary(GalbaMigrants)

# Family: bernoulli

# Links: mu = logit

# Formula: InfectionStatus ~ NZMSBiomassMigrants + Size

# Data: Galba (Number of observations: 161)

# Samples: 4 chains, each with iter = 2000; warmup = 1000; thin = 1;

# total post-warmup samples = 4000

#

# Population-Level Effects:

# Estimate Est.Error l-95% CI u-95% CI Rhat Bulk_ESS Tail_ESS

# Intercept -3.43 1.29 -6.05 -0.99 1.00 3530 2133

# NZMSBiomassMigrants -0.00 0.01 -0.01 0.01 1.00 4579 3114

# Size 0.28 0.11 0.08 0.50 1.00 3705 2302

#

# Samples were drawn using sampling(NUTS). For each parameter, Bulk_ESS

# and Tail_ESS are effective sample size measures, and Rhat is the potential

# scale reduction factor on split chains (at convergence, Rhat = 1).

**Testing dilution effect in specific trematode taxa in the native snail *Pyrgulopsis*.**

**Trematodes in the Superfamily Pronocephaloidea (coded mono because make monostome cercariae)with migrants:**

PyrgMonoMigrants <- brm(MonoStatus ~ (1|CageSize) + (1|year) + NZMSBiomassMigrants+Size, data=Pyrg, family="bernoulli", control = list(adapt_delta=0.99))

plot(PyrgMonoMigrants)

summary(PyrgMonoMigrants)

# Family: bernoulli

# Links: mu = logit

# Formula: MonoStatus ~ (1 | CageSize) + (1 | year) + NZMSBiomassMigrants + Size

# Data: Pyrg (Number of observations: 417)

# Samples: 4 chains, each with iter = 2000; warmup = 1000; thin = 1;

# total post-warmup samples = 4000

#

# Group-Level Effects:

# ~CageSize (Number of levels: 2)

# Estimate Est.Error l-95% CI u-95% CI Rhat Bulk_ESS Tail_ESS

# sd(Intercept) 0.98 1.08 0.02 3.92 1.00 1095 1258

#

# ~year (Number of levels: 2)

# Estimate Est.Error l-95% CI u-95% CI Rhat Bulk_ESS Tail_ESS

# sd(Intercept) 1.16 1.22 0.04 4.38 1.00 1189 1162

#

# Population-Level Effects:

# Estimate Est.Error l-95% CI u-95% CI Rhat Bulk_ESS Tail_ESS

# Intercept -5.96 1.51 -8.87 -2.94 1.00 1421 1757

# NZMSBiomassMigrants 0.01 0.00 0.00 0.02 1.00 4653 2707

# Size 1.01 0.19 0.65 1.38 1.00 2523 2501

#

# Samples were drawn using sampling(NUTS). For each parameter, Bulk_ESS

# and Tail_ESS are effective sample size measures, and Rhat is the potential

# scale reduction factor on split chains (at convergence, Rhat = 1).

**Trematodes in the Superfamily Pronocephaloidea (coded mono because make monostome cercariae) without migrants:**

PyrgMonoNoMigrants <- brm(MonoStatus ~ (1|Rep) + (0 + Treatment|Rep)+(1|CageSize) + (1|year) + Treatment + Size, data=Pyrg, family="bernoulli", control = list(adapt_delta=0.99))

#no errors!

plot(PyrgMonoNoMigrants)

summary(PyrgMonoNoMigrants)

# Family: bernoulli

# Links: mu = logit

# Formula: MonoStatus ~ (1 | Rep) + (0 + Treatment | Rep) + (1 | CageSize) + (1 | year) + Treatment + Size

# Data: Pyrg (Number of observations: 417)

# Samples: 4 chains, each with iter = 2000; warmup = 1000; thin = 1;

# total post-warmup samples = 4000

#

# Group-Level Effects:

# ~CageSize (Number of levels: 2)

# Estimate Est.Error l-95% CI u-95% CI Rhat Bulk_ESS Tail_ESS

# sd(Intercept) 1.04 1.16 0.03 4.07 1.00 1791 1646

#

# ~Rep (Number of levels: 8)

# Estimate Est.Error l-95% CI u-95% CI Rhat Bulk_ESS Tail_ESS

# sd(Intercept) 0.17 0.15 0.01 0.57 1.00 2364 2086

# sd(Treatment) 0.14 0.12 0.01 0.46 1.00 1776 2052

#

# ~year (Number of levels: 2)

# Estimate Est.Error l-95% CI u-95% CI Rhat Bulk_ESS Tail_ESS

# sd(Intercept) 1.19 1.24 0.05 4.45 1.00 1737 2142

#

# Population-Level Effects:

# Estimate Est.Error l-95% CI u-95% CI Rhat Bulk_ESS Tail_ESS

# Intercept -5.96 1.59 -9.21 -2.79 1.00 2737 2153

# Treatment 0.22 0.15 -0.07 0.51 1.00 3970 2598

# Size 1.01 0.19 0.65 1.39 1.00 4381 3093

#

# Samples were drawn using sampling(NUTS). For each parameter, Bulk_ESS

# and Tail_ESS are effective sample size measures, and Rhat is the potential

# scale reduction factor on split chains (at convergence, Rhat = 1).

**Trematodes in the Superfamily Allocreadiidea (coded Xiph because make xiphiocercariae) with migrants:**

PyrgXiphMigrants <- brm(XiphStatus ~ (1|CageSize) + (1|year) + NZMSBiomassMigrants + Size, data=Pyrg, family="bernoulli", control = list(adapt_delta=0.99))

#no errors!

plot(PyrgXiphMigrants)

summary(PyrgXiphMigrants)

#

# Family: bernoulli

# Links: mu = logit

# Formula: XiphStatus ~ (1 | CageSize) + (1 | year) + NZMSBiomassMigrants + Size

# Data: Pyrg (Number of observations: 417)

# Samples: 4 chains, each with iter = 2000; warmup = 1000; thin = 1;

# total post-warmup samples = 4000

#

# Group-Level Effects:

# ~CageSize (Number of levels: 2)

# Estimate Est.Error l-95% CI u-95% CI Rhat Bulk_ESS Tail_ESS

# sd(Intercept) 0.99 1.20 0.03 4.13 1.00 1411 1868

#

# ~year (Number of levels: 2)

# Estimate Est.Error l-95% CI u-95% CI Rhat Bulk_ESS Tail_ESS

# sd(Intercept) 1.71 1.38 0.27 5.42 1.00 1434 1344

#

# Population-Level Effects:

# Estimate Est.Error l-95% CI u-95% CI Rhat Bulk_ESS Tail_ESS

# Intercept 0.13 1.68 -3.06 3.55 1.00 1714 1790

# NZMSBiomassMigrants 0.00 0.00 -0.01 0.01 1.00 4771 2861

# Size -0.19 0.19 -0.58 0.17 1.00 2618 2227

#

# Samples were drawn using sampling(NUTS). For each parameter, Bulk_ESS

# and Tail_ESS are effective sample size measures, and Rhat is the potential

# scale reduction factor on split chains (at convergence, Rhat = 1).

**Trematodes in the Superfamily Allocreadiidea (coded Xiph because make xiphiocercariae) without migrants**

PyrgXiphNoMigrants <- brm(XiphStatus ~ (1|Rep) + (0 + Treatment|Rep)+ (1|CageSize) + (1|year) + Treatment + Size, data=Pyrg, family="bernoulli", control = list(adapt_delta=0.99))

#no errors!

plot(PyrgXiphNoMigrants)

summary(PyrgXiphNoMigrants)

# Family: bernoulli

# Links: mu = logit

# Formula: XiphStatus ~ (1 | Rep) + (0 + Treatment | Rep) + (1 | CageSize) + (1 | year) + Treatment + Size

# Data: Pyrg (Number of observations: 417)

# Samples: 4 chains, each with iter = 2000; warmup = 1000; thin = 1;

# total post-warmup samples = 4000

#

# Group-Level Effects:

# ~CageSize (Number of levels: 2)

# Estimate Est.Error l-95% CI u-95% CI Rhat Bulk_ESS Tail_ESS

# sd(Intercept) 0.98 1.24 0.02 4.08 1.00 1527 1859

#

# ~Rep (Number of levels: 8)

# Estimate Est.Error l-95% CI u-95% CI Rhat Bulk_ESS Tail_ESS

# sd(Intercept) 0.46 0.31 0.03 1.20 1.00 1133 1304

# sd(Treatment) 0.28 0.22 0.01 0.81 1.01 1357 1815

#

# ~year (Number of levels: 2)

# Estimate Est.Error l-95% CI u-95% CI Rhat Bulk_ESS Tail_ESS

# sd(Intercept) 1.66 1.34 0.29 5.04 1.00 1884 2113

#

# Population-Level Effects:

# Estimate Est.Error l-95% CI u-95% CI Rhat Bulk_ESS Tail_ESS

# Intercept 0.28 1.77 -2.92 4.04 1.00 2380 2336

# Treatment -0.12 0.20 -0.49 0.29 1.00 3082 2655

# Size -0.19 0.20 -0.58 0.19 1.00 4293 2763

#

# Samples were drawn using sampling(NUTS). For each parameter, Bulk_ESS

# and Tail_ESS are effective sample size measures, and Rhat is the potential

# scale reduction factor on split chains (at convergence, Rhat = 1).
